# LONELINESS AND SELF-EFFICACY AMONG INDIVIDUALS WITH CHRONIC CONDITIONS: THE MEDIATING ROLE OF DEPRESSION

**DOI:** 10.1093/geroni/igae098.3017

**Published:** 2024-12-31

**Authors:** Barbara Dabrowski, Christine McKibbin, Catherine Carrico, Laurance Goodwin, Stacy Carling, Elizabeth Punke, Abby Teply, Sabine Schenck

**Affiliations:** University of Wyoming, Laramie, Wyoming, United States; University of Wyoming, Laramie, Wyoming, United States; University of Wyoming, Laramie, Wyoming, United States; University of Wyoming, Laramie, Wyoming, United States; University of Wyoming, Laramie, Wyoming, United States; University of Wyoming, Laramie, Wyoming, United States; University of Wyoming, Laramie, Wyoming, United States; University of Wyoming, Laramie, Wyoming, United States

## Abstract

Approximately 60% of Americans live with one or more chronic conditions (CDC, 2023). Chronic disease self-management programs empower individuals to take an active role in their health management. Self-efficacy is an important construct determining self-management behaviors. Recently, low self-reported ratings of self-efficacy for managing chronic disease have been associated with high loneliness ratings (Lee et al., 2023); however, other variables that may be important regarding the relationship between loneliness and self-efficacy are less known. Depressive symptoms are associated with both loneliness and management of chronic disease. This study examined the mediating role of depressive symptoms in the relationship between loneliness and self-efficacy among individuals with chronic disease participating in the Self-Management Resource Centers, Chronic Disease Self-Management Program (CDSMP). The sample (n = 48, M = 63.0 years old, SD = 18.0) comprised primarily White females (n = 37; 77%) who presented with chronic conditions and completed a CDSMP workshop. Regression analyses revealed that baseline loneliness predicted baseline depressive symptoms (β =.49, p <.001). Both baseline loneliness (β = -.46, p <.006) and depressive symptoms (β = -.75, p <.001) predicted baseline self-efficacy. The relationship between loneliness and self-efficacy became non-significant (β = -.12, p =.301) after controlling for depressive symptoms. Examination of loneliness and depression to changes in self-efficacy pre- to post-intervention showed similar results. Depressive symptoms are important in the relationship between loneliness and self-efficacy among participants in the CDSMP. Addressing depressive symptoms may support improvement in self-efficacy among CDSMP participants experiencing loneliness.

